# Could Tumor Necrosis Factor Serve as a Marker for Cardiovascular Risk Factors and Left Ventricular Hypertrophy in Patients with Early-Onset Coronary Artery Disease?

**DOI:** 10.3390/diagnostics14040449

**Published:** 2024-02-18

**Authors:** Marta Białecka, Violetta Dziedziejko, Krzysztof Safranow, Andrzej Krzystolik, Zuzanna Marcinowska, Dariusz Chlubek, Monika Rać

**Affiliations:** 1 Department of Internal Diseases and Hematology, Military Medical National Research Institute, Szaserów 128, 04-349 Warszawa, Poland; m.bialeckaaa@gmail.com; 2 Department of Biochemistry, Pomeranian Medical University, Powstańców Wielkopolskich 72, 70-111 Szczecin, Poland; viiola@pum.edu.pl (V.D.); chrissaf@mp.pl (K.S.); zuzanna.marcinowska@pum.edu.pl (Z.M.); dchlubek@pum.edu.pl (D.C.); 3 Department of Cardiology, County Hospital in Szczecin, Arkońska 4, 71-455 Szczecin, Poland; akrzystolik@poczta.onet.pl

**Keywords:** TNF, metabolic syndrome, dyslipidemia, left ventricular hypertrophy, CAD risk

## Abstract

**Introduction:** Tumor necrosis factor (TNF), a pro-inflammatory cytokine, can be produced by cardiomyocytes, leading to metabolic disorders in the myocardium. The objective of this study was to assess the relationship between plasma levels of the TNF cytokine and the presence of known biochemical and clinical risk factors for cardiovascular disease, along with the parameters of cardiac morphology in patients diagnosed with coronary artery disease (CAD) at a young age. **Materials and Methods:** The study group included 75 men aged up to 50 years and 25 women aged up to 55 years. The plasma TNF concentration was measured by use of the ELISA assay. Echocardiography and electrocardiographic examinations were performed in all patients. **Results:** We observed positive correlations for TNF with the BMI ratio, weight, waist and hip circumference. We also found negative correlations for TNF with HDL levels and ApoA concentrations, and positive correlations with the ApoB/ApoA1 ratio, Apo B, IL6, LDL and TG concentrations. These results suggest an association between higher plasma TNF concentrations and components of metabolic syndrome, including dyslipidemia. TNF may be a potential risk factor for impaired diastolic function. **Conclusions:** While TNF may be useful for diagnosing certain risks in CAD patients, the TNF measurement cannot be used as a surrogate test for echocardiography.

## 1. Introduction

Coronary artery disease (CAD) has become a major public health challenge. It is associated with high morbidity and mortality worldwide and is increasingly prevalent in the Western world [1]. Although the disorder usually affects older people, the last few decades have seen an increasing incidence of CAD among younger people. The manifestation of CAD at a young age is referred to as premature or early-onset CAD, but various studies consider the upper age limit to be between 35 and 55 years [2]. In young patients with acute coronary incidents, revascularization is associated with low mortality. However, mortality in this group increases after an extended period. Those with premature CAD have an overall poor prognosis; patients die within 15 years of their first episode. A history of myocardial infarction (MI), diabetes, smoking and lower ejection fraction puts them at risk for significantly higher mortality [3]. A better understanding of this group of patients is important in the era of preventive cardiology. Understanding the importance of oxidative stress markers for endothelial dysfunction and inflammation in the pathophysiology of early-onset CAD may help define new aspects of vascular biology and additional markers of future risk. Both primary and secondary prevention plays pivotal roles in averting coronary events.

Inflammation is increasingly seen as a new modifiable cardiovascular risk factor [4]. One of the interesting pro-inflammatory cytokines is the TNF protein. It is a cachectin, or cachexin, produced mainly by activated monocytes and macrophages and in much smaller amounts by other cells, such as granulocytes, endothelial cells, adipocytes, keratinocytes, fibroblasts, neutrophils, mast cells, T lymphocytes and smooth muscle myocytes [5]. From a biochemical point of view, TNF is a glycoprotein composed of 182 amino acids, formed through the post-translational modification of a 212-amino acid polypeptide. There are also shorter or longer isoforms produced by tissues [6]. The human TNF gene is located on chromosome 6 at position p21.3 and contains four exons. The last exon shows 56% similarity to the LTA (lymphotoxin alpha, TNF-β) gene, also encoding a secretory protein. The 3′ UTR of TNF contains the ARE (AU-rich element). TNF affects cells by binding to the appropriate receptors on the cell membrane surface. To date, two types of such receptors have been identified: TNF-R1 and TNF-R2. These receptors have been found on immunocompetent cells, among others [7,8]. The stimulation of these receptors stimulates cells to produce and release cytokines and activates the arachidonic acid cascade, which leads to an increase in the intracellular free radical concentration, leads to cell death (apoptosis), stimulates the liver to produce acute-phase proteins, including CRP, increases insulin resistance in peripheral tissues and attracts neutrophils [9,10,11,12]. Therefore, TNF is an established pro-atherosclerotic factor, but the mechanism is not entirely understood.

As a pro-inflammatory cytokine, TNF may also be produced by cardiomyocytes, which typically occurs under unfavorable hemodynamic conditions with excessive pressure or volume load [13]. It may lead to metabolic disorders in the myocardium, left ventricular dysfunctions, reduced peripheral flow and left ventricular remodeling. The role of TNF in insulin resistance has been better studied [14] than the role of TNF in atherosclerosis. For instance, there is a lack of information about TNF in patients with early-onset CAD. A better understanding of this group of patients is important for preventive cardiology. Therefore, the objective of this study was to assess the relationship between plasma levels of the TNF cytokine and the presence of known biochemical and clinical risk factors for cardiovascular disease, along with the parameters of cardiac morphology in patients diagnosed with coronary artery disease (CAD) at a young age.

## 2. Materials and Methods

### 2.1. Characteristics of the Study Group

The research was approved by the bioethics committee of the Pomeranian Medical University (resolution no. BN-001/162/04, approval dated 6 November 2017). All studies were conducted in accordance with the Declaration of Helsinki. Informed consent to participate in the study was obtained from all patients. These consents, along with the complete study records of each patient, are kept in the Department of Biochemistry at the PUM.

The criterion for inclusion in the study was the fulfillment of one of three conditions:

1. A history of myocardial infarction;
2. Angiographically documented coronary stenosis (involving ≥50% of the left main stem coronary artery or ≥70% of the branches);
3. A history of myocardial revascularization surgery (CABG or PTCA).

However, subjects with a recent history of acute coronary syndrome, significant heart failure or heart defect (NYHA ≥ II), severe renal (serum creatinine > 3 mg/dL) or liver failure, type 1 diabetes, thyroid disorders, rheumatoid arthritis or cancer were excluded from the study. Consequently, 100 consecutive, criterion-eligible, clinically stable patients from the Cardiology Department of the Regional Hospital in Szczecin were included in the study. The study group with diagnosed coronary artery disease consisted of 75 men aged up to 50 years and 25 women aged up to 55 years. Due to people only with strong risk factors developing early-onset CAD, age was an inclusion criterion for the study. All subjects received a drug treatment for cardiac reasons. Cases of acute coronary syndrome were excluded from this study. Additionally, each patient included in the study was required to be a minimum of 30 days after a potential revascularization procedure or coronary angiography.

### 2.2. Diagnostic Tests

#### 2.2.1. Physical Examination

Morphometric measurements (height, weight and waist and hip circumference) and blood pressure by the RR method were taken in all patients. The body mass index (BMI), waist-to-hip ratio (WHR) and mean arterial pressure were calculated.

#### 2.2.2. Biochemical Tests

Blood sampling for biochemical and genetic tests and other diagnostic tests were performed no earlier than 30 days after any revascularization procedure or cardiovascular episode. Blood was collected from fasting forearm veins at the Central Laboratory of the Independent Public Clinical Hospital No. 1 in Szczecin, Poland, into vacuum tubes (Sarstedt), 2.7 mL per EDTA for the blood morphology evaluation and 4.6 mL per clot for serum determinations. Standard automated methods, including a Sysmex analyzer for blood counts and a Roche Cobas 6000 analyzer for assessing the serum levels of total cholesterol, HDL and LDL fractions, TG, apolipoproteins (ApoA1, ApoB and Lp(a)), glucose and C-reactive protein (hsCRP), were employed for the analysis.

#### 2.2.3. Testing Plasma TNF Levels by ELISA Method

Blood was collected on EDTA, centrifuged for 10 min at 4000× *g* and the plasma was then stored at −80 °C. The TNF concentration in plasma was determined using an ELISA method with a commercially available immunoenzymatic assay (EIAab, Wuhan EIAab Science Co., Ltd., Wuhan, China). The sample preparation procedure for measuring the concentration of the parameters under study was carried out by developing our own ELISA assay methodology based on the manufacturer’s enclosed protocol and our own published ELISA assay methodology [15]. The zero standard (0 ng/mL) was prepared using a sample diluent from the ELISA kit, while the highest concentration standard was dissolved in 1.0 mL of the diluent to form a 10.0 ng/mL solution. Patient plasma samples were diluted 20-fold with the sample diluent. Throughout the procedure, careful attention was paid to ensure that the total time for dispensing reagents into the test plate did not exceed 10 min. After the final wash, the plate was inverted and placed on clean paper towels to remove any residual wash buffer. An automated ELX 808IU Microplate Reader (Bio-Tek Instruments Inc., Winooski, VT, USA, Agilent Technologies, Santa Clara, CA, USA) calibrated adequately with recombinant human protein concentrations in the appropriate range was used to determine the concentration of the proteins tested in plasma. The absorbance of the test samples was read against the calibration curve at 490 nm. The assays were evaluated and the minimum detectable dose (MDD) of TNF ranged from 0.038 to 0.191 pg/mL. The mean MDD was 0.106 pg/mL. The MDD was determined by adding two standard deviations to the mean optical density value of twenty zero-standard replicates and calculating the corresponding concentration. Similarly, the sCD36, I16, VEGF and PCSK9 plasma protein concentrations were determined using the same available immunoenzymatic assays (EIAab, Wuhan EIAab Science Co., Ltd., Wuhan, China).

#### 2.2.4. Echocardiography Examination

Echocardiography was conducted on the patients using a Medison SA 9900 machine, performed by a specialist cardiologist. The anatomical parameters were evaluated, including the LV end-diastolic and end-systolic diameter and volume, aortic diameter, left atrial diameter, end-diastolic thickness of the interventricular septum and posterior LV wall and right ventricular end-diastolic dimension. The biplane Simpson method recommended by the European Society of Cardiology was used to calculate the left ventricular ejection fraction [16]. The left ventricular mass was calculated using the Devereux equation [17]. The left ventricular mass index was calculated by dividing the LVM by the body surface area [18]. To assess the left ventricular diastolic function (LVDF), we used conventional echocardiographic imaging (a measurement of the maximum early diastolic inflow velocity (E) and late diastolic inflow velocity (A), from which the E-to-A quotient was calculated) and pulsed-wave tissue Doppler imaging (TDI) (a measurement of the early diastolic motion velocity (E′) and late diastolic motion velocity (A′) in the lateral and septal parts of the mitral annulus, from which the E′-to-A′ quotient was calculated). Values of E/A from 1 to 2.5 and E′/A′ > 1, values of diastolic dysfunction E/A < 1 and E′/A′ < 1, values of pseudonormalization E/A from 1 to 2.5 and E′/A′ < 1 and values of restriction E/A > 2.5 and E′/A′ > 1 were used as the criteria for determining normal diastolic function [19].

#### 2.2.5. Electrocardiographic Examination

A standard 12-lead electrocardiogram at rest was performed. Heart rate and regularity, atrioventricular and intraventricular conduction, cardiac axis, the width of the QRS complexes, R and S amplitudes, QT and PQ intervals, ST segment height and features of past myocardial infarctions were evaluated. The electrocardiography evaluation criteria were based on the standards in Poland [20].

### 2.3. Statistical Methods

In most cases, the distributions of the parameters of measurable clinical characteristics were significantly different from usual (the Shapiro–Wilk test), so non-parametric tests were used in the calculations, including the Kruskal–Wallis test, followed by the Mann–Whitney U test. To assess the significance of correlations between quantitative variables, Spearman’s rank correlation coefficient was used. The multiple linear regression analysis was performed to determine independently significant TNF concentration predictors. The classic Bonferroni correction was applied in the significance analysis. As a total of 128 statistical associations (including correlations and comparisons in the whole group and in the subgroups) were analyzed in the manuscript, we applied the Bonferroni-corrected threshold *p*-value equal to 0.05/128 = 0.00039. Statistical calculations were performed using Statistica 6.1 software.

## 3. Results

The characteristics of the study CAD group, along with information on past cardiac procedures and treatments used, are presented in Table 1. Data on the clinical, biochemical, echocardiography and electrocardiography patients’ parameters are presented in Table 2. As anticipated, differences in clinical parameters between the male and female subgroups were identified; these results are detailed in another paper [21].

Patients were categorized into groups based on the presence or absence of metabolic syndrome and ventricular hypertrophy. Following the guidelines from the European Society of Cardiology [22], left ventricular hypertrophy (LVH) was diagnosed when the LVMI exceeded >125 g/m^2^ in men and >110 g/m^2^ in women. Metabolic syndrome was defined as the simultaneous presence of a BMI > 30 and at least one of two conditions: diabetes or hypertension. The Mann–Whitney U test indicated borderline significance (*p* = 0.092) when comparing cases with and without metabolic syndrome and those with and without left ventricular hypertrophy (*p* = 0.0054). However, after applying the Bonferroni correction, no statistical significance was observed.

Male gender is a known predisposing factor for coronary artery disease, which was reflected in the high percentage of men among the patients studied. Therefore, to verify the possible influence of the patients’ gender on the results obtained, an analysis was performed to divide patients with CAD into subgroups of men and women. Statistically significant correlations between the TNF serum concentration and quantitative variables assessed with the Spearman rank correlation coefficient in the early-onset CAD patients’ group and in the subgroups of males and females are presented in Table 3. Only the statistically significant or borderline correlations were presented for better clarity of the results. An exact test indicated an association between higher TNF and impaired LVDF (*p* = 0.00083). The Bonferroni correction for multiple comparisons was applied to the significance analysis, with a resulting value of 0.00039. In the classical calculation, statistically significant correlations were found for plasma TNF levels with various morphometric parameters (BMI, weight and waist and hip circumference), biochemical parameters (hsCRP, sCD36, HDL, LDL, triacylglycerols, Lp(a), ApoA1, ApoB and ApoB/ApoA1) and with cardiography parameters (left ventricular end-diastolic diameter, left atrium diameter, E/A ratio, right ventricular end-diastolic diameter, LVMI, left ventricular end-diastolic and systolic volumes, QRS II width, QRS V5 width, RV5(6) and RV5(6) + SV1(2) amplitudes). However, none of these analyzed parameters remained statistically significant after applying the Bonferroni correction. Of all the analyzed parameters, only the correlation TNF with the ApoB/ApoA1 ratio remained significant after the Bonferroni correction. In cardiology, this index is considered a recommended predictor of CAD risk compared to lipid profile values [23]. It could be complemented by the determination of TNF. Multiple linear regression analyses revealed that a significant independent predictor of a higher plasma TNF level was a higher ApoB/ApoA ratio (Table 4).

## 4. Discussion

### 4.1. TNF and Morphometry Parameters

Others demonstrated that mean TNF values in blood serum were significantly higher in patients with hypertension. Also, patients with a history of myocardial infarction or stroke exhibited a higher serum TNF concentration than patients without those complications in their medical history [24]. Liu et al. [25] assessed the plasma level of TNF as an apoptotic marker in acute myocardial infarction patients and found increased levels in older people (≥65 years) compared to the younger group (<65 years) after 24 h and 3 and 5 days after a clinical percutaneous coronary intervention. However, that level was lower over time in both patient groups. The increased secretion of IL-6 is also known to be a response to increased TNF levels and both TNF and IL-6 have been shown to be present in higher concentrations in children with diabetes or risk factors for diabetes. A larger body mass index is also associated with a high grade of TNF in that group [26,27]. One study suggests a pathogenic role of the TNF system in the development of cardiovascular disease in T1DM. Of all the evaluated inflammatory proteins in that study, only TNF was positively associated with some of the evaluated blood pressure indexes in T1DM patients, even after adjusting for potential confounders and after 14 years of diagnoses [28]. In contrast, no significant differences were found between the groups for serum in TNF concentrations in the overweight and obese groups compared to the non-obese group [29,30,31,32] and also between women and men groups in type 2 diabetes patients [33]. However, in another study [34], TNF was positively associated with the BMI index and waist circumference. In the blood of the morbidly obese, patients were found to have significantly elevated levels of TNF, but a decreased level of high-density lipoprotein (HDL) cholesterol, compared with the healthy individuals. The treatment resulted in over a 9.4% reduction in body weight, but did not decrease the TNF concentrations [35]. TNF could, therefore, be considered an independent cardiovascular risk factor in metabolic syndrome [36]. On the other hand, one research work [37] revealed IL-6 as an independent predictor of type 2 diabetes, not TNF, while another demonstrated [38] that hsCRP is a more sensitive marker associated with obesity than IL-6 and TNF.

In our study, the serum TNF level was 1.33 ± 0.36 pg/mL and was significantly higher in the male (1.37 ± 0.37 pg/mL) than the female group (1.23 ± 0.29 pg/mL, *p* = 0.049). Different authors report different levels of plasma TNF concentrations. The range varies from a few [39] to several tens of pg/mL [40] in people with CAD and approximately 2 pg/mL in healthy people [41]. Statins reduce plasma TNF levels [42], and our patients were all treated with statins. We observed positive correlations between TNF and the BMI ratio, weight and waist and hip circumference in patients with early-onset CAD. All these parameters are components of metabolic syndrome.

### 4.2. TNF and Lipids

The typical constellation of biochemical abnormalities that characterize heart failure increases angiotensin-converting enzyme activity, increases oxidative stress and raises endothelin-1 levels, leading to a reduction in endothelium-derived nitric oxide (NO) bioavailability and increased vasoconstrictor tone. In addition, the increase in circulating pro-inflammatory cytokines in heart failure patients, notably, TNF, may also reduce the synthesis of NO by downregulating the expression of endothelial nitric oxide synthase, the key enzyme involved in NO production. TNF also affects the lipid metabolism and hypertriglyceridemia by decreasing lipoprotein lipase activity in adipocytes [43]. It is known [44,45] that TNF promotes atherosclerosis by increasing LDL transcytosis across endothelial cells, thereby facilitating LDL retention in vascular walls through NF-κB and PPAR-γ activation. In human peripheral blood mononuclear cells, HDL inhibited TNF release and this inhibitory effect was specific for HDL and was not affected by low-density lipoprotein or very-low-density lipoprotein [46]. Patel et al. reported that, TNF negatively correlated with HDL and Apo A1 in a group of CAD patients, but not with LDL and the ApoA/ApoB ratio [47]. The multivariate regression analysis indicated that the inverse association between the level of HDL and TNF and the occurrence of ischemic cerebral vascular disease was statistically significant. That is why the authors suggested that the combination test of HDL and TNF could raise the accuracy of that disease diagnosis [48,49].

We also found negative correlations between TNF and HDL levels and ApoA concentrations. Positive correlations were observed between TNF and the ApoB/ApoA1 ratio, Apo B, IL6, LDL and TG concentrations. Our results suggest an association of higher TNF plasma concentrations with metabolic syndrome components, including dyslipidemia. It should be noted that TNF production in monocytes from patients treated with atorvastatin is reduced [50]. However, in our study, almost all patients were treated with statins, so the influence on the results was relatively mild.

### 4.3. TNF and Echocardiography

Not much data have been published thus far analyzing the association between variations in TNF and echocardiography parameters. TNF was inversely correlated with the E/A ratio [51]. In the atrial fibrillation group of patients, TNF levels were significantly correlated with left atrial volume [52]. The left atrial volume index is a relatively robust measure of diastolic function, as it is less acutely load-sensitive than various Doppler-derived indexes.

In our study, positive correlations were found between TNF and parameters that could reflect left ventricular enlargement, left ventricular end-diastolic and systolic diameter and volume, left atrium diameter and the left ventricular mass index (LVMI). Some correlations were not significant in the subgroups of males and females, suggesting a confounding effect of gender. For example, the correlation with the right ventricular mean systolic pressure seemed accidental, because it was not similar in the gender subgroups to the whole study group and was not reflected in other echocardiographic results. However, our results suggested that a higher plasma TNF concentration was associated with a higher risk of left ventricular hypertrophy and could be a potential risk factor for impaired diastolic function in patients with early-onset CAD.

### 4.4. TNF and Electrocardiographic Examination

In all inflammatory conditions, marked increases in cytokines, in particular, are seen [53]. In the study by Nikolic et al. [54], the correlation between TNF levels and heart rate (HR) was not significant. TNF levels were significantly and inversely correlated with heart rate variability in the lower values group. Szewieczek et al. showed in their study [55] that the QRS amplitude correlated negatively and QTc interval correlated positively with the TNF serum level. The TNF level correlated inversely with % of ejection fraction [56] and directly with the extent of the prolongation of the QT interval [57], even in patients without heart failure. TNF causes this by stimulating ROS. It was shown that the chance of dying could be predicted by the extent of the elevations of TNF as IL6 also. Whether these cytokines are causative factors or merely markers is unknown, but studies have demonstrated [58] that TNF reduces myocardial contractility in patients with heart failure through disturbances of intracellular calcium homeostasis, suggesting a causative relationship for altered myocardial function, at least in heart failure. The “electrical storm” of recurrent ventricular arrhythmia seen with cardiac disease is also associated with elevated levels of cytokines and other inflammatory markers [57]. Overall, the TNF level marked at 48 h after the cardiovascular episode has a sensitivity of 78% and a specificity of 72.5% in predicting a cardiovascular ischemic event such as angina, reinfarction, HF and death [59,60].

In our study, LV hypertrophy was assessed using both echo and ECG examinations to facilitate comparisons with previous studies. However, the echo criteria of both LV and RV hypertrophy are more sensitive and specific than the ECG criteria. We found a more frequent tendency for impaired left ventricle diastolic function (LVDF) and a higher E/A ratio in patients with a higher TNF level. We also observed a tendency for a longer QTc II interval and width, which may also be associated with features of left ventricular hypertrophy. Other results were not reflected in that tendency. In our study, a higher TNF level was associated with lower RV5(6) and RV5(6) + SV1(2) parameters, which is the opposite to left ventricular hypertrophy.

The evaluation of cardiac parameter variability is presently a research tool of determined clinical utility, but much more useful could be the coincidental evaluation of an additional test parameter, such as the level of cytokines in plasma. Chronic low-grade inflammation and its early detection of inflammation may facilitate meaningful lifestyle changes in the CAD high-risk group. ELISA is a simple method widely used in most labs, while serum is an easily obtained material from humans [61]. However, the results of our study did not provide clear answers. None of the correlation coefficient (Rs) values exceeded 0.5, indicating a limitation in the potential of using the TNF measurement to elucidate the pathomechanism of CAD. Consequently, the study results did not support the justification of employing the TNF measurement as a surrogate test for echocardiography. The study also had some limitations. A study limitation was the lack of a randomly selected control group consisting of healthy individuals (without CAD) with the same exclusion criteria as the study group, including age and sex. This consideration should be taken into account in future research. Another limitation of the study was that, by its nature, a cross-sectional study cannot draw causal conclusions. It was, therefore, difficult to decide whether TNF was the cause, risk factor or end-result of other factors, such as metabolic syndrome and elevated cholesterol.

## 5. Conclusions

TNF, as a pro-inflammatory cytokine, may be useful in the prediction of some biochemical cardiovascular risk factors in CAD patients, but the TNF measurement cannot be used as a surrogate test for echocardiography.

## Figures and Tables

**Table 1:** The characteristics of the study CAD group (number of patients—100).

Parameter	Value
Gender (% males)	75%
Age of patients (years)	49.9 ± 5.91
Past MI	70%
Age of the first MI (years)	44.0 ± 5.6
Time since diagnosis of MI to joining the program (years)	3.20 ± 0.74
History of hypertension	66%
Age at diagnosis of hypertension (years)	42.6 ± 8.6
Past PTCA	71%
Past CABG	37%
Past smoking	89%
Years smoking	18.9 ± 9.8
Diabetes type 2	13%
Statins	96%
Anti-platelet drugs (Aspirin)	90%
ACEI	80%
Beta-blockers	88%
Diuretics	31%
ARB	17%
Calcium channel blockers	18%

MI—myocardial infarction; PTCA—percutaneous transluminal coronary angioplasty; CABG—coronary artery bypass grafting; ACEI—angiotensin-1-converting enzyme inhibitor; ARB—angiotensin 2 receptor blocker.

**Table 2:** The clinical, biochemical, echocardiography and electrocardiography patients’ parameters.

Parameter	CAD *n* = 100
BMI (kg/m^2^)	28.1 ± 3.98
WHR	0.96 ± 0.09
Weight (kg)	83.4 ± 17.0
Waist (cm)	98.3 ± 12.5
MAP (mmHg)	93.8 ± 9.35
Systolic BP (mmHg)	127 ± 14.2
Diastolic BP (mmHg)	77.0 ± 9.01
Glucose (mg/dL)	107 ± 24.8
hsCRP (mg/L)	1.82 ± 2.7
WBC (G/L)	6.80 ± 0.22
Platelets (G/L)	218 ± 44.6
MPV (fL)	10.6 ± 0.09
Hemoglobin (g/dL)	14.8 ± 1.14
Hematocrit (%)	43.9 ± 3.17
RBC (T/L)	4.91 ± 0.42
MCV (fL)	89.6 ± 4.40
Total cholesterol (mg/dL)	173 ± 40.4
LDL cholesterol (mg/dL)	102 ± 36.2
HDL cholesterol (mg/dL)	48.4 ± 11.5
ApoA1 (mg/dL)	154 ± 38.4
ApoB/ApoA1	0.53 ± 0.15
Lp(a) (mg/dL)	40.3 ± 49.3
Triacylglycerols (mg/dL)	136 ± 57.1
TNF (pg/mL)	1.33 ± 0.36
IL-6 (pg/mL)	1.69 ± 2.77
VEGF (pg/mL)	236 ± 17.2
PCSK9 (ng/mL)	358 ± 10.7
sCD36 (µg/mL)	15.78 ± 12.9
LVEF [%]	53.6 ± 11.1
LVMI [g/m^2^]	183 ± 62.3
Left ventricular end-diastolic diameter [mm]	51.3 ± 7.17
Left ventricular end-diastolic volume [mL]	121 ± 43.4
Left atrium diameter [mm]	38.6 ± 5.71
LVDF normal	38%
LVDF impaired	54%
LVDF pseudonormal	8%
Right ventricular end-diastolic diameter [mm]	32.9 ± 5.60
Right ventricular mean systolic pressure [mmHg]	22.0 ± 6.27
DT [ms]	221 ± 69.5
E/A ratio	1.12 ± 0.37
Tissue Doppler E′ [cm/s]	10.1 ± 11.0
Heart rate [1/min]	70.7 ± 12.1
PQ interval [s]	0.19 ± 0.10
QRS II width [s]	0.081 ± 0.020
QRS V5 width [s]	0.083 ± 0.038
RV5(6) amplitude [mm]	12.0 ± 6.04
SV1(2) amplitude [mm]	8.66 ± 4.56
RV1(2) amplitude [mm]	2.65 ± 2.58
SV5(6) amplitude [mm]	3.08 ± 3.25
RV1(2) + SV5(6) amplitude [mm]	5.70 ± 4.29
RV5(6) + SV1(2) amplitude [mm]	20.5 ± 8.30
QTc II interval [s]	0.40 ± 0.04
QTc V4 interval [s]	0.41 ± 0.04
Electrical axis deviation	6%
ECG criteria of past myocardial infarction	58%
ST depression	31%

BMI—body mass index; WHR—waist-to-hip ratio; MAP—mean arterial pressure; WBC—white blood cell; MPV—mean platelet volume; RBC—red blood cell; MCV—mean corpuscular volume; TNF—tumor necrosis factor α; IL6—interleukin 6; VEGF—vascular endothelial growth factor; PCSK9—proprotein convertase subtilisin/kexin type 9; sCD36—soluble CD36 protein; LVEF—left ventricular ejection fraction; LVMI—left ventricular mass index; LVDF—left ventricle diastolic function; DT—deceleration time.

**Table 3:** The correlations between circulating TNF concentration (pg/mL) and quantitative parameters of early-onset CAD patients in the whole study group and in the subgroups of males and females (statistically significant value with Bonferroni correction for multiple comparisons is 0.00039).

Parameter	Correlations for CAD Patients (*n* = 100)		Correlations for Males (*n* = 75)		Correlations for Females (*n* = 25)	
	Rs	*p*-Value	Rs	*p*-Value	Rs	*p*-Value
BMI (kg/m^2^)	0.27	0.011	0.24	0.048	0.23	0.29
Weight (kg)	0.30	0.0045	0.29	0.018	0.07	0.75
Waist (cm)	0.28	0.009	0.30	0.016	0.18	0.43
Hip (cm)	0.32	0.0031	0.27	0.031	0.46	0.037
Platelets (G/L)	0.16	0.13	0.09	0.48	0.47	0.019
hsCRP (mg/L)	0.23	0.029	0.24	0.048	0.31	0.15
sCD36 (µg/mL)	−0.25	0.016	−0.19	0.13	0.06	0.77
IL-6 (pg/mL)	0.17	0.099	0.18	0.14	0.14	0.52
HDL-cholesterol (mg/dL)	−0.32	0.0016	−0.29	0.015	−0.14	0.53
LDL-cholesterol (mg/dL)	0.23	0.024	0.23	0.054	0.21	0.33
Triacylglycerols (mg/dL)	0.33	0.0014	0.37	0.002	0.07	0.76
Lp(a) (mg/dL)	−0.32	0.0016	0.02	0.87	−0.21	0.32
ApoA1 (mg/dL)	−0.28	0.0061	−0.28	0.022	−0.04	0.85
ApoB (mg/dL)	0.23	0.026	0.26	0.033	0.10	0.63
ApoB/ApoA1	0.44	0.00012	0.44	0.00015	0.25	0.23
Left ventricular end-diastolic diameter [mm]	0.33	0.0019	0.39	0.0013	−0.18	0.60
Left ventricular end-systolic diameter [mm]	0.18	0.099	0.26	0.037	−0.22	0.34
Left atrium diameter [mm]	0.25	0.020	0.14	0.25	0.46	0.031
E/A ratio	0.32	0.0020	0.30	0.015	0.51	0.018
Right ventricular end-diastolic diameter [mm]	0.22	0.039	0.17	0.18	0.31	0.17
LVMI [g/m^2^]	0.23	0.037	0.24	0.059	0.04	0.86
Left ventricular end-diastolic volume [mL]	0.30	0.0045	0.32	0.010	0.04	0.86
Left ventricular end-systolic volume [mL]	0.23	0.033	0.26	0.040	0.07	0.77
QRS II width [s]	0.22	0.037	0.18	0.14	0.30	0.18
QRS V5 width [s]	0.33	0.0020	0.37	0.0026	0.09	0.70
R_V5(6)_ amplitude [mm]	−0.29	0.0077	−0.37	0.0032	−0.24	0.29
R_V5(6)_ + S_V1(2)_ amplitude [mm]	−0.26	0.016	−0.40	0.0012	−0.06	0.95
S_V1(2)_ amplitude [mm]	−0.08	0.47	−0.26	0.045	0.56	0.0064
QTc II interval [s]	0.20	0.062	0.34	0.0057	−0.14	0.54
QTc V4 interval [s]	0.17	0.13	0.33	0.010	−0.23	0.30
Right ventricular mean systolic pressure [mmHg]	0.18	0.095	0.25	0.047	−0.053	0.81

**Table 4:** Multiple linear regression model with logarithmically transformed plasma TNF concentration as dependent variable.

Independent Variables	β Coefficient (95%CI)	*p*-Value
ApoB/ApoA1 ratio	+0.37	0.00026

## Data Availability

The full study records of each patient are kept in the Department of Biochemistry at the Pomeranian Medical University.
